# Machine Learning Prediction for Functional Impairment, Falls, and Fractures in Postmenopausal Women

**DOI:** 10.1093/geroni/igaf122.3621

**Published:** 2025-12-31

**Authors:** Junya Uchida

**Affiliations:** University of Toronto, Toronto, Ontario, Canada

## Abstract

Machine learning-based prediction models have gained support for their robustness and flexibility, particularly with complex data common in geriatrics research. They may be well-suited to studying complex aging questions, such as predicting functional status among postmenopausal women. The objective of this study is to develop an interactive prediction model for prevalent, clinically relevant, geriatric health outcomes: functional impairment, falls, and fractures. We simulated a dataset (n = 20,000) to emulate baseline characteristics of the Women’s Health Initiative (WHI) study population, a large study of postmenopausal women. The simulated dataset had a mean age of 63.2 years (SD: 7.25), 82.4% non-Hispanic White. Maximum follow-up was 25 years. We examined random survival forest, XGBoost, and Lasso-Cox models. Models were trained and compared across multiple domain-specific feature sets comprising demographic, socioeconomic, behavioral, functional, and health-related features, selected from existing evidence, with time-dependent ROC-AUC, calibration, and Brier score. SHapley Additive exPlanations (SHAP) guided feature selection. The random survival forest outperformed other models in predicting functional impairment, falls, and fractures. Reduced models incorporating 30 features demonstrated comparable performance relative to the comprehensive models with 57 features, while improving computational efficiency. These reduced models demonstrated strong discrimination (ROC-AUCs >0.80) and predictive accuracy (Brier scores < 0.10) across most time points and were subsequently deployed in the interactive application. This study demonstrates the utility of machine learning-based prediction to guide targeted interventions by capturing the complex, heterogeneous nature of geriatric health outcomes. Deploying such advanced models into a user-friendly application may support data-driven decision-making in geriatric clinical practice.

